# Active Gallbladder Lavage Using a Double‐pigtail Plastic Stent Delivery System During Endoscopic Ultrasound‐guided Gallbladder Drainage (With Video)

**DOI:** 10.1002/deo2.70247

**Published:** 2025-11-18

**Authors:** Tesshin Ban, Yoshimasa Kubota, Kei Ando, Naoto Imura, Youta Hirayama, Shun Sasoh, Tomoaki Ando, Takashi Joh

**Affiliations:** ^1^ Department of Gastroenterology and Hepatology Gamagori City Hospital Aichi Japan

**Keywords:** acute cholecystitis, drainage, endosonography, gallbladder, therapeutic irrigation

## Abstract

Endoscopic ultrasound‐guided gallbladder drainage (EUS‐GBD) with a lumen‐apposing metallic stent is advised for frail patients with acute cholecystitis. However, concerns remain about severe tissue reactions and potential negative impacts on subsequent surgery. EUS‐GBD using a double‐pigtail plastic stent (DPPS) is an alternative for these patients. However, multiple steps harbor bile or air leaking from the anastomosis site. Some DPPS systems comprise an inner sheath that mounts a DPPS and a delivery pusher catheter. This DPPS system gains a lavage function, similar to a long plastic irrigation tube, when the inner sheath is retracted to the “halfway back position” within the DPPS. This single‐arm retrospective study reviewed the active gallbladder lavage technique using this “halfway back position” of the DPPS system during EUS‐GBD for patients with acute cholecystitis who left the option of laparoscopic cholecystectomy (Lap‐C) as a final option, depending on clinical and performance status recovery. The technical success rate was 97.3% (36/37). The clinical success rate among those with technical success was 100% (36/36). The median total duration was 25 min 7 s, which included a lavage duration of 8 min 18 s. The volume of lavage was 40 mL, and the number of attempts was two. The length of the anastomosis measured using computed tomography was 11.6 mm. No adverse events were reported. Nine patients (25.0%) were scheduled for elective Lap‐C, and none required conversion to open surgery. The active gallbladder lavage technique using the DPPS system during EUS‐GBD was both feasible and acceptable.

## Graphical abstract

Endoscopic ultrasound‐guided gallbladder drainage using a double‐pigtail plastic stent (DPPS) harbors the risk of bile leakage and pneumoperitoneum through the anastomosis site. When the inner sheath of the DPPS system is retracted halfway back into the stent, multiple side holes emerge on the stent end, which accelerates the active gallbladder lavage. This active and straightforward gallbladder lavage using the DPPS system resulted in a high technical and clinical success rate, and developed neither bile leakage nor pneumoperitoneum.

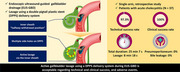

## 1 Introduction

In terms of clinical benchmarks such as efficacy and safety, endoscopic ultrasound‐guided gallbladder drainage (EUS‐GBD) using a lumen‐apposing metallic stent (LAMS) is recommended over the standard percutaneous or transpapillary approaches in fragile patients with acute cholecystitis [1, 2]. However, EUS‐GBD with a double‐pigtail plastic stent (DPPS) can be chosen when percutaneous or transpapillary approaches are unsuitable, the LAMS is unavailable or not approved, and laparoscopic cholecystectomy (Lap‐C) is still a final option. However, concerns about bile leakage at the anastomosis site and pneumoperitoneum remain during EUS‐GBD with DPPS [3, 4, 5]. EUS‐GBD using DPPS comprises the following steps: gallbladder needle puncture is performed, followed by guidewire insertion, tract dilation, and DPPS indwelling [4, 5]. Gallbladder lavage can be performed using either a puncture needle or through additional catheters. The multistep procedure carries the risk of bile or air leakage and pneumoperitoneum at the anastomosis site [5]. Meanwhile, some authors advocate gallbladder aspiration, followed by lavage via a thinner, long plastic tube during endoscopic trans‐papillary gallbladder drainage (ETGBD) [6, 7]. However, no well‐designed study has investigated the aforementioned potential complications for EUS‐GBD with lavage via the long tube [4, 5, 8]. Furthermore, detachment of the contracted gallbladder from the duodenum may interfere with placement of the second guidewire and/or DPPS.

Therefore, we present a novel gallbladder lavage technique during the EUS‐GBD procedure using a double‐pigtail plastic stent delivery system, which functions as a long irrigation tube [9]. Herein, we aim to evaluate this groundbreaking lavage technique in a case series.

## 2 Procedure/Technique

### 2.1 Study Design and Patients

This single‐arm study was conducted retrospectively by reviewing electronic medical records from June 2023 to April 2025 at a single center, Gamagori City Hospital. It was approved by the Ethics Committee of Gamagori City Hospital (approval number 274‐3; approval date: June 2, 2025).

The inclusion criteria was primarily based on the Tokyo Guidelines 18 as follows [10, 11]: patients diagnosed with moderate to severe acute cholecystitis who were considered high‐risk for emergency cholecystectomy according to age‐adjusted Charlson's comorbidity index (ACCI) and American Society of Anesthesiologists physical status (ASA‐PS); Surgically high‐risk patients with mild cholecystitis who required gallbladder drainage due to persistent severe pain and worsening inflammation despite conservative treatment, as well as surgically eligible patients with mild cholecystitis who clinically deteriorated as described above but lacked available surgeons. For patients with mild to moderate cholecystitis, scores of ACCI ≥ 6 and ASA‐PS ≥ 3 were considered surgical risk factors. For patients with severe cholecystitis, ACCI ≥ 4 and ASA‐PS ≥ 3 indicated that they were ineligible for surgery. Based on the patients’ written informed consent, which included three drainage approaches—EUS‐GBD, percutaneous, and transpapillary—the cohort patients chose EUS‐GBD over the percutaneous or transpapillary approach.

Exclusion criteria included: patients with contraindications for endoscopic intervention (e.g., respiratory or cardiac decompensation, shock, and severe coagulopathy), surgically altered gastric anatomy, concurrent acute cholangitis (ETGBD was prioritized), gallbladder tumorous lesions or ascites seen on cross‐sectional images, and a physical status of irreversible ASA‐PS ≥ 4. The possibility of Lap‐C had been considered for all patients based on their clinical and performance status recovery.

### 2.2 Active Gallbladder Lavage Technique Using a DPPS Delivery System During EUS‐GBD

**FIGURE 1:**
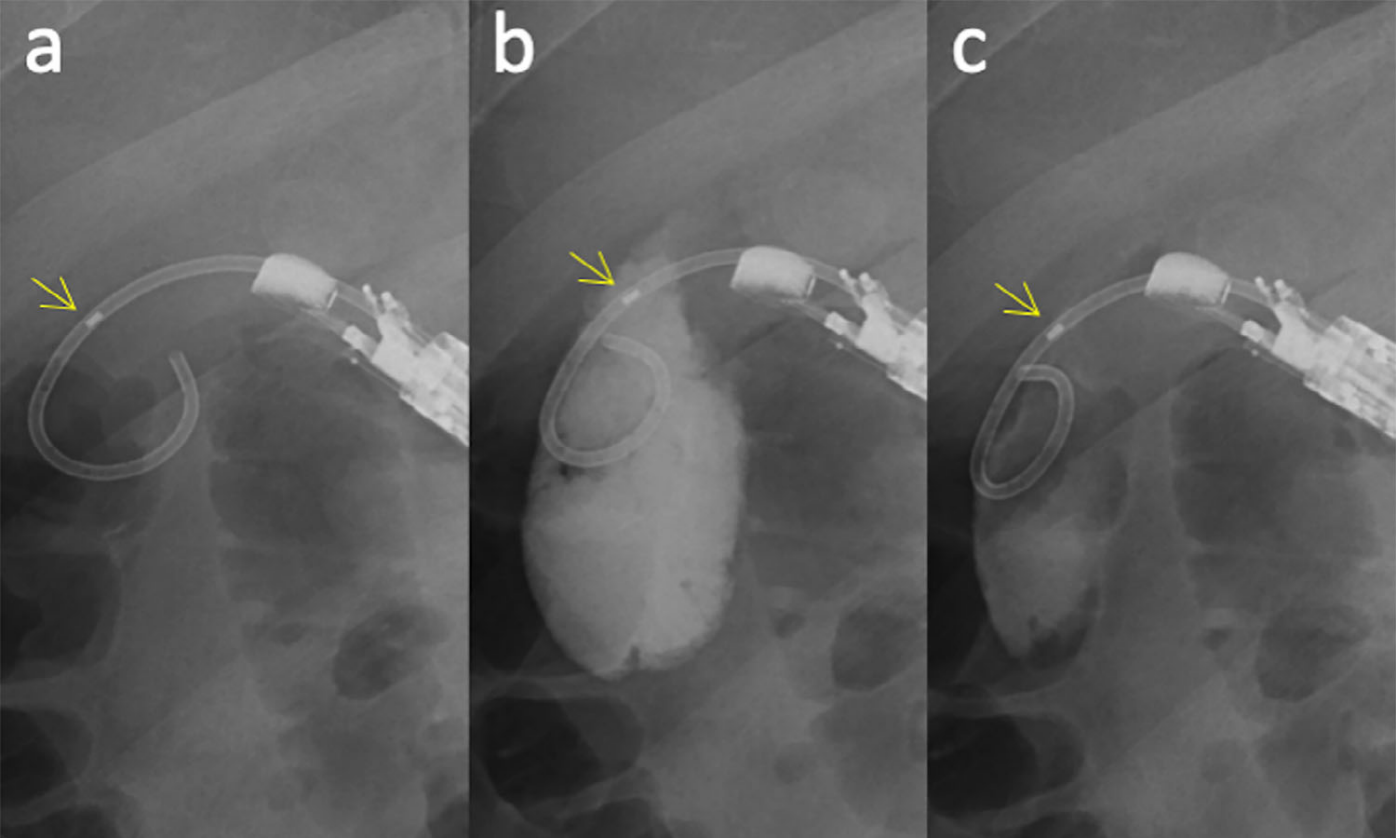
Gallbladder lavage using a DPPS delivery system during EUS‐GBD. (a) After removing a guidewire, the inner sheath is retracted to the halfway‐withdrawal position within the DPPS, and multiple holes emerge in the distal pigtailed area. At this position, the gallbladder contents are sufficiently aspirated from the inner sheath. (b) Contrast‐containing saline is injected via the inner sheath. (c) The next aspiration. The lavage is repeated until the aspirated bile changes into serous. The yellow arrows indicate the radiopaque marker at the tip of the inner sheath. DPPS, double‐pigtail plastic stent; EUS‐GBD, endoscopic ultrasound‐guided gallbladder drainage.

EUS‐GBD was performed with the patient in the prone position under moderate sedation using benzodiazepines (midazolam, 0.03 mg/kg) and opioid receptor agonists (pentazocine, 15.0 mg/body) administered intravenously. Additional midazolam (1.0 mg) was injected, as required, to maintain a Ramsey score of 3 or 4 [12]. The procedure was performed as described in previous reports, but only by endoscopists skilled in interventional echoendosonography or under their supervision. Briefly, oblique‐viewing linear endoscopic ultrasonography (EG‐580UT and ultrasonography processor SU‐1; Fujifilm, Tokyo, Japan) was used to visualize the neck of the gallbladder via the duodenal bulb or stomach, followed by gallbladder puncture using a 19‐gauge lancet needle (SonoTip Pro Control; Medi‐Globe GmbH, Rohrdorf, Germany), 0.025‐inch hydrophilic guidewire (VisiGlide2; Olympus, Tokyo, Japan) insertion and coiling, electrocautery dilation (Cysto‐Gastro‐Sets, 6‐Fr; Endo‐flex GmbH, Voerde, Germany), and advancement of the DPPS system (e.g., Advanix J, 7‐ Fr, 4/7/10 cm; Boston Scientific, Marlborough, US) [4, 5]. This system consists of the DPPS connected to a delivering pusher and mounted over an inner sheath. After DPPS system advancement, the guidewire was removed and the inner sheath was retracted to the halfway‐withdrawal position within the DPPS (Figure 1a) (Video S1). In this position, the gallbladder contents were aspirated from the end of the inner sheath using a 50‐mL syringe set on an inflation/deflation device (Alliance II integrated inflation/lithotripsy device; Boston Scientific) and repeated gallbladder lavage with contrast‐containing saline until the aspirated bile became serous (Figure 1b, c) (Video S1) [9]. The saline lavage volume was set as the first aspiration minus 10 mL. Finally, the inner sheath was removed for stent deployment (Video S1).

**FIGURE 2:**
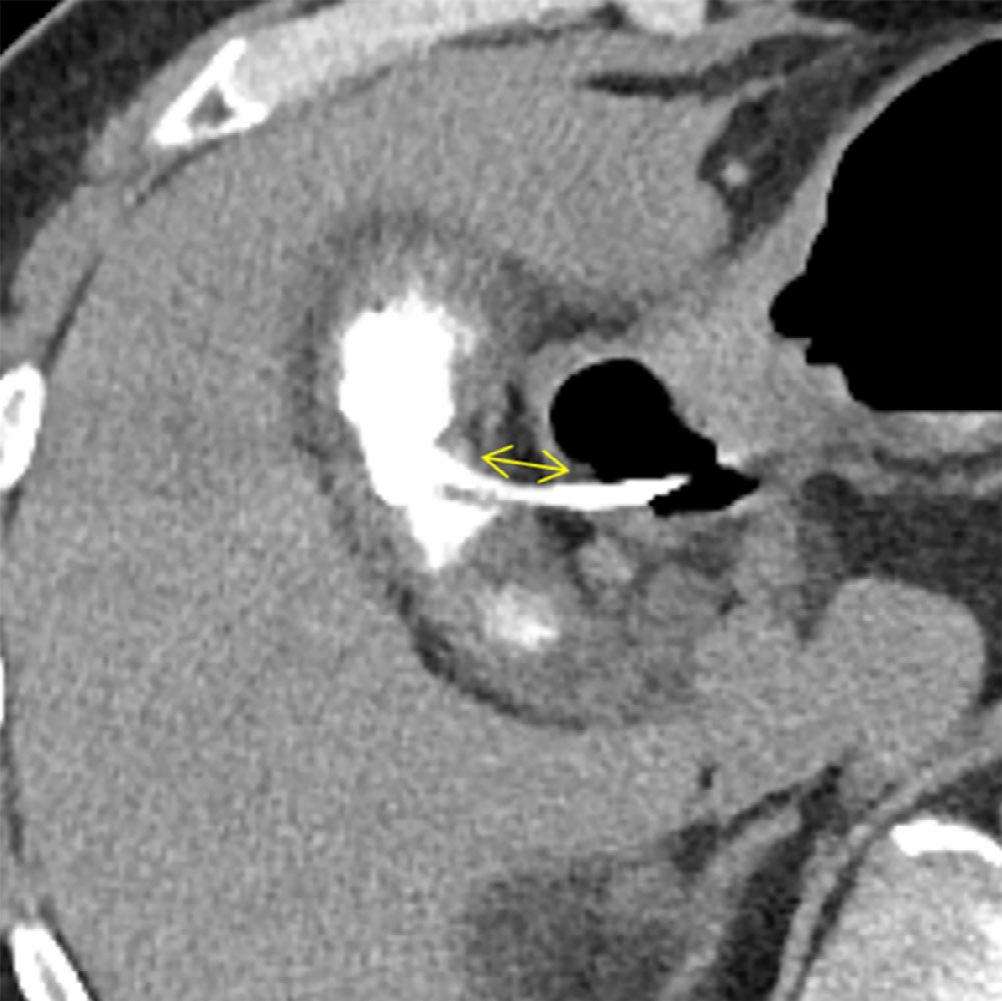
Surveillance for pneumoperitoneum, bile leakage, and length of anastomosis site on post‐procedural CT following EUS‐GBD. Post‐gallbladder lavage was performed twice with 40 mL of contrast‐containing saline. No air or bile leakage is observed around the gallbladder after the procedure. The yellow double‐headed arrow indicates the length of the anastomosis site, measured as the gap between the center of the gallbladder wall and the opposite duodenum. CT, computed tomography; EUS‐GBD, endoscopic ultrasound‐guided gallbladder drainage.

Consecutive post‐procedural fluoroscopy and abdominal computed tomography (CT) were performed for surveillance of the stent position, bile leakage, and pneumoperitoneum (Figure 2). Laboratory inflammatory findings, including white blood cell (WBC) count and C‐reactive protein (CRP), were measured immediately before, 3 h after, and one day after the index procedure. Subsequently, inflammatory marker surveillance was performed every few days.

### 2.3 Definition of Variables

The patient's background, laboratory inflammatory findings, details of the acute cholecystitis and procedure, and consequences were abstracted. Postprocedural CT findings included pneumoperitoneum, contrast medium leakage, and anastomosis site length. The length of the anastomosis site on CT was measured as the gap between the center of the gallbladder wall and the opposite gastrointestinal wall (Figure 2). The diagnosis, severity, and surgical risk assessments were based on the Tokyo Guidelines 2018 [10, 11]. The total procedural duration refers to the time from when the scope is inserted through the mouth until the DPPS detaches from the inner sheath. Duration, excluding lavage, refers to the net time of EUS‐GBD. The lavage duration was defined as the interval between the halfway‐withdrawal position of the inner sheath within the DPPS and its detachment. Technical success was defined as aspiration or rinsing once or more using the DPPS system. Clinical success was defined as resolution of clinical symptoms and laboratory findings associated with cholecystitis without the need for further intervention [4]. Adverse events (AEs) were defined and graded according to the American Society for Gastrointestinal Endoscopy criteria [13]. This study only focused on early AEs occurring within 14 days of the procedure. Conversion to open surgery during Lap‐C was also recorded.

### 2.4 Outcomes

We assessed technical and clinical success, contrast medium leakage, and pneumoperitoneum. Additionally, we investigated the puncture site, 7 Fr‐DPPS length, total duration, net duration of EUS‐GBD, lavage duration/volume/frequency, biliary bacterial culture, anastomosis length, time to afebrile state, time to peak of WBC and CRP levels, progression of cholecystitis severity, and the interval of antibiotic treatment, open conversion in elective Lap‐C, and other AEs.

### 2.5 Statistical Analysis

This was not a comparative study; therefore, we only performed descriptive statistics expressing nominal variables as numbers with ratios, and ordinal/continuous variables as medians with interquartile ranges (IQRs).

## 3 Results

The baseline characteristics of the 37 patients are summarized in Table 1. Twenty‐four (64.9%) patients were male, and the median age was 85 years (IQR: 78–91 years). Approximately 90% of the patients had moderate‐to‐severe acute cholecystitis. The etiologies of the acute cholecystitis cases were gallstones (32/37 [86.5%]), a fully covered self‐expandable metal stent for biliary obstruction due to pancreatic cancer (4/37 [10.8%]), and a plastic stent placed for the treatment of biliary cancer (1/37 [2.7%]). WBC count, CRP, ACCI, ASA‐PS, and onset to drainage were 12,700/µL (IQR: 9000–15,700), 16.6 mg/dL (IQR: 10.6–23.6), score of 6 (IQR: 5–7), score of 3 (IQR: 2–3), and 3 (IQR: 2–5) days, respectively. Antiplatelets/anticoagulants were prescribed to 12 (32.4 %) patients. We attempted EUS‐GBD on aspirin in 18.9% of cases (7/37 patients) and on cilostazol in 10.8% of cases (4/37 patients). The direct oral anticoagulant (rivaroxaban) was stopped on the morning of the EUS‐GBD in one patient (2.7%). Rivaroxaban was restarted on day 5 because subsequent blood tests confirmed stable hemoglobin and serum blood urea nitrogen levels.

**TABLE 1:** Baseline characteristics of the patients with acute cholecystitis, *N* = 37.

Age	Years	Median (IQR)	85	(78–91)
Sex	Man	Number (%)	24	(64.9)
Severity	Mild/moderate/severe	Number (%)	3/29/5	(8.1/78.4/13.5)
Etiology	Gallstone/FCSEMS/plastic stent	Number (%)	32/4/1	(86.5/10.8/2.7)
WBC	10^3^/µL	Median (IQR)	12.7	(9.0–15.7)
CRP	mg/dL	Median (IQR)	16.6	(10.6–23.6)
Antiplatelet/anticoagulant	Yes	Number (%)	12	(32.4)
	Aspirin/cilostazol/rivaroxaban	Number (%)	7/4/1	(18.9/10.8/2.7)
ACCI	Score	Median (IQR)	6	(5–7)
ASA‐PS	Score	Median (IQR)	3	(2–3)
Onset to drainage	Days	Median (IQR)	3	(2–5)

Abbreviations: ACCI, age‐adjusted Charlson's comorbidity index; ASA‐PS, American Society of Anesthesiologists physical status; CRP, C‐reactive protein; FCSEMS, fully covered self‐expandable metal stent; IQR, interquartile range; WBC, white blood cell count.

The results of active lavage using the DPPS system during EUS‐GBD are summarized in Table 2. The technical success rate was 97.3% (36/37 patients). The clinical success rate among those with technical success was 100% (36/36 patients); the time to become afebrile was 1 day (IQR: 1–2), and the times to peak of WBC and CRP levels were 0 (IQR: 0–0) and 1 day (IQR: 0–1), respectively. The grade of cholecystitis in none of the patients worsened further. Antibiotics were administered for 7 days (IQR: 5–9) in this cohort. A total of 34 of the 36 technically successful attempts (94.4%) were achieved from the duodenal bulb, whereas the remaining two (5.6%) were achieved from the stomach. A 7‐Fr DPPS with a length of 4 or 7 cm was used (35/36 patients; 97.2%). The median total duration of EUS‐GBD was 25 min 7 s (IQR: 21 min 23 s–27 min 54 s), which included a lavage duration of 8 min 18 s (IQR: 6 min 45 s–10 min 51 s). Thus, the net time for EUS‐GBD was 16 min 14 s (IQR: 12 min 53 s–18 min 53 s). The volume and frequency of lavage were 40 mL (IQR: 30–50 mL) and two attempts (IQR: 2–3 attempts). Positive biliary bacterial cultures were observed in 88.9% (32/36 patients). The length of the anastomosis measured using CT was 11.6 mm (IQR: 8.4–15.8 mm). No pneumoperitoneum or contrast medium leakage was observed on the fluoroscopic and subsequent CT images. No other early AEs were reported. Elective Lap‐C was attempted in 9/36 patients (25.0%), and none of them underwent conversion to open cholecystectomy. However, elective Lap‐C was avoided in 27/36 patients (75.0%) due to high surgical risk and poor life expectancy.

**TABLE 2:** Results of active lavage using the double‐pigtail plastic stent (DPPS) system during endoscopic ultrasound‐guided gallbladder drainage (EUS‐GBD), *N* = 37.

Technical success		Yes	Number (%)	36	(97.3)
Clinical success		Yes	Number (%)	36	(100) *
Time to afebrile state		Days	Median (IQR)	1	(1–2) *
Time to peak	WBC	Days	Median (IQR)	0	(0–0) *
	CRP	Days	Median (IQR)	1	(0–1) *
Deterioration of the severity grade		Yes	Number (%)	0	0 *
Interval of antibiotics		Days	Median (IQR)	7	(5–9) *
Puncture site		Duodenal bulb/stomach	Number (%)	34/2	(94.4/5.6) *
Length of 7 Fr‐DPPS		4/7/10 cm	Number (%)	18/17/1	(50.0/47.2/2.8) *
Duration	Total	min s	Median (IQR)	25 min 7 s	(21 min 23s–27 min 54 s) *
	Excluding lavage	min s	Median (IQR)	16 min 14 s	(12 min 53 s–18 min 53 s) *
	For lavage	min s	Median (IQR)	8 min 18 s	(6 min 45 s–10 min 51 s) *
Lavage	Volume	mL	Median (IQR)	40	(30–50) *
	Attempts	Number	Median (IQR)	2	(2–3) *
Biliary bacterial culture		Positive	Number (%)	32	(88.9) *
Length of the anastomosis		mm	Median (IQR)	11.6	(8.4–15.8) *
Contrast‐medium leakage		Yes	Number (%)	0	0 *
Pneumoperitoneum		Yes	Number (%)	0	0 *
Other AEs		Yes	Number (%)	0	0 *
Elective Lap‐C		Ineligible/as planned/ open conversion	Number (%)	27/9/0	(75.0/25.0/0) *

Abbreviations: AEs, adverse events; CRP, C‐reactive protein; DPPS, double‐pigtailed plastic stent; EUS‐GBD, endoscopic ultrasonography‐guided gallbladder drainage; IQR, interquartile range; Lap‐C, laparoscopic cholecystectomy; min, minutes; s, seconds; WBC, white blood cell count; *, one case of EUS‐GBD failure was excluded from the analysis.

In the single patient with failed EUS‐GBD due to a previous duodenal bulb metallic stent, a later attempt using a forward‐viewing linear echoendoscope salvaged this failure.

## 4 Discussion

We previously reported technical tips for the active gallbladder lavage technique using a DPPS delivery system during EUS‐GBD. In this method, when the inner sheath is withdrawn halfway back in the DPPS, multiple side holes emerge on the distal pigtailed area in addition to the single hole on the stent end, and function as a long plastic tube [9]. This setup safely replaces the highly viscous infected bile with serous saline through repeated aspiration and lavage. This potentially reduces the risk of infected bile leaking into the abdominal cavity via the anastomosis before the fistula matures and prevents incomplete drainage caused by thick bile. In the gallbladder aspiration phase, continuous suction from the scope channel enhances the bile aspiration effect through the inner sheath; thereby, this maneuver reduces contamination of intraduodenal gas via the slit between the DPPS and the pusher.

In this study, EUS‐GBD with active lavage using the DPPS system for acute cholecystitis was mainly performed in advanced‐aged, fragile patients with a median age of 85 years, ACCI score of 6, and ASA‐PS score of 3. Clinical success was achieved in all patients, with rapid recovery of inflammatory markers, including fever, WBC, and CRP, except for one patient with poor maneuverability of the puncture needle due to a previous metallic stent inside the duodenal bulb. EUS‐GBD combined with lavage using the DPPS system required a median duration of 25 min 7 s to complete the entire procedure, including 8 min 18 s for two rinses. Comparing our results with a similar previous report evaluating EUS‐GBD using a 7‐Fr DPPS combined with a long plastic tube [14], the technical and clinical success rates (97.3% and 100% in the present study vs. 95.7% and 95.5%) were excellent in both. Noticeably, the total procedure in our method took a shorter time than theirs (median, 25 min 7 s vs. 40 min), even though we included the time for the intensive lavage (8 min 18 s); this may be because their method required more steps and devices. No unfavorable effects, including bile leakage‐related AEs, developed after our method; this was confirmed with a thorough review of post‐procedural CT images. In contrast, bile peritonitis or intraperitoneal abscess developed in 0% and 8.7% (2/23) in the previous studies, which included patients managed with long plastic tube drainage. Of note, pneumoperitoneum treated with conservative management occurred in 6.7% (2/30) and 21.7% (5/23) of cases reported previously [8, 14]. The gap between the dilated fistula and the thinner stent, the space between double stents within the same fistula, and multiple device insertion after the dilation can potentially exacerbate intra‐ and post‐procedural air and bile leaks. To overcome these leakages, EUS‐GBD using electrocautery‐enhanced LAMS is recommended as an alternative intervention for surgically high‐risk patients with acute cholecystitis [2, 5]. However, this device is unaffordable and has not been globally approved for the treatment of acute cholecystitis.

This was a small‐sample‐size, single‐arm, retrospective cohort study; therefore, the method should be validated with a larger, multicenter, comparative study. The limitations and future perspectives of this study are as follows: First, further evaluation based on precise criteria, such as bile aspiration speed, volume, and bile retrieval rate, is needed to reach definitive conclusions, as these parameters depend on the gallbladder contents, including necrotizing debris, clots, and sludge or microstones. These residues may block the inner sheath of the DPPS system, which is originally designed with a bore diameter to pass the 0.035‐inch guidewire. If the inner sheath obstruction persists despite saline purging, we must abandon the gallbladder lavage and only perform stent deployment. Second, the risk of bile leakage and pneumoperitoneum remains, even though the 7‐Fr DPPS potentially seals the anastomosis site after 6‐Fr dilation. Because dilation with the 6‐Fr electrocautery device can cause a burning effect that may expand the anastomosis site beyond 7‐Fr if used for extended electrocautery, physicians should keep the electrocautery dilator pressed steadily against the walls to push it forward quickly. Third, a comparative study for EUS‐GBD with and without gallbladder lavage is warranted to highlight the clinical advantages and disadvantages of gallbladder rinsing. In particular, long‐term outcomes beyond 14 days, such as stent dysfunction, should be examined in patients who undergo lavage followed by permanent DPPS placement and compared to those without lavage. Permanent DPPS placement is performed out of necessity in the surgically high‐risk population of advanced age. Fourth, there may be a negative impact on the Lap‐C maneuver; in previous studies conducted in a small number of patients, EUS‐GBD using a long plastic tube with or without a 7‐Fr DPPS does not seem to affect surgical outcomes [8, 14]. Therefore, the Lap‐C would be safely completed without technical difficulties in patients undergoing EUS‐GBD using the DPPS [5]. EUS‐GBD using electrocautery‐enhanced LAMS was reported not to preclude patients from eventually undergoing Lap‐C, as the rates of surgical success and conversion from laparoscopic to open cholecystectomy were similar to those with percutaneous drainage [15]. Notably, pericholecystic adhesions and/or fistula created by the electrocautery‐enhanced LAMS interfered with Lap‐C, which inevitably led to open surgery in all three cases [16]. LAMS has a large diameter and strong lumen apposing effect for connecting the gallbladder and duodenum, which leads to significant tissue reaction, and eventually buried LAMS syndrome [17]. This tissue reaction may later complicate maneuverability during Lap‐C at the anastomosis site. Therefore, the feasibility of Lap‐C after EUS‐GBD with LAMS continues to be a topic of ongoing debate. In contrast, in EUS‐GBD using DPPS, a 7 Fr plastic stent with a small diameter and no lumen‐apposing effect is placed at the anastomosis site. A plastic stent might be more bio‐friendly, hypothetically compared to LAMS. However, future studies should be designed to compare the surgical outcomes between patients who undergo EUS‐GBD with LAMS and those with DPPS deployed using our method.

In conclusion, active gallbladder lavage through the halfway withdrawn inner sheath during EUS‐GBD with the DPPS system is potentially acceptable in terms of technical and clinical success, and early AEs profile. Further comparative and well‐designed studies are needed to validate our technique.

## Author Contributions

**Tesshin Ban** and **Yoshimasa Kubota**: Study concept, endoscopic procedures, manuscript writing, and video editing. **Kei Ando**, **Naoto Imura**, **Youta Hirayama**, **Shun Sasoh**, and **Tomoaki Ando**: Manuscript revision. **Takashi Joh**: Manuscript revision and final approval of the manuscript. All authors critically revised the manuscript, approved the final version for publication, and assume responsibility for all aspects of the study.

## Conflicts of Interest

The authors declare no conflicts of interest.

## Funding

The authors received no specific funding for this work.

## Ethics Statement

This retrospective study was approved by the Ethics Committee of Gamagori City Hospital (approval number 274‐3; approval date: June 2, 2025).

## Consent

Written informed consent for EUS‐GBD, including alternatives like transpapillary drainage, percutaneous drainage, and surgery, was obtained from all patients.

## Supporting information




**Supporting Video 1**: Active gallbladder lavage technique using a double‐pigtail plastic stent delivery system during endoscopic ultrasound‐guided gallbladder drainage.
